# Disrupted Functional Connectivity of the Anterior Cingulate Cortex in Cirrhotic Patients without Overt Hepatic Encephalopathy: A Resting State fMRI Study

**DOI:** 10.1371/journal.pone.0053206

**Published:** 2013-01-07

**Authors:** Long Jiang Zhang, Rongfeng Qi, Jianhui Zhong, Ling Ni, Gang Zheng, Jian Xu, Guang Ming Lu

**Affiliations:** 1 Department of Medical Imaging, Jinling Hospital, Clinical School of Medical College, Nanjing University, Nanjing, China; 2 Department of Biomedical Engineering, Zhejiang University, Hangzhou, Zhejiang, China; Hangzhou Normal University, China

## Abstract

**Background:**

To evaluate the changes of functional connectivity of the anterior cingulate cortex (ACC) in patients with cirrhosis without overt hepatic encephalopathy (HE) using resting state functional MRI.

**Methodology/Principal Findings:**

Participants included 67 cirrhotic patients (27 minimal hepatic encephalopathy (MHE) and 40 cirrhotic patients without MHE (non-HE)), and 40 age- and gender- matched healthy controls. rsfMRI were performed on 3 Telsa scanners. The pregenual ACC resting-state networks (RSNs) were characterized by using a standard seed-based whole-brain correlation method and compared between cirrhotic patients and healthy controls. Pearson correlation analysis was performed between the ACC RSNs and venous blood ammonia levels, neuropsychological tests (number connection test type A [NCT-A] and digit symbol test [DST]) scores in cirrhotic patients. All thresholds were set at *P*<0.05, with false discovery rate corrected. Compared with controls, non-HE and MHE patients showed significantly decreased functional connectivity in the bilateral ACC, bilateral middle frontal cortex (MFC), bilateral middle cingulate cortex (MCC), bilateral superior temporal gyri (STG)/middle temporal gyri (MTG), bilateral thalami, bilateral putamen and bilateral insula, and increased functional connectivity of bilateral precuneus and left temporo-occipital lobe and bilateral lingual gyri. Compared with non-HE patients, MHE showed the decreased functional connectivity of right MCC, bilateral STG/MTG and right putamen. This indicates decreased ACC functional connectivity predominated with the increasing severity of HE. NCT-A scores negatively correlated with ACC functional connectivity in the bilateral MCC, right temporal lobe, and DST scores positively correlated with functional connectivity in the bilateral ACC and the right putamen. No correlation was found between venous blood ammonia levels and functional connectivity in ACC in cirrhotic patients.

**Conclusions/Significance:**

Disrupted functional connectivity in ACC was found in cirrhotic patients which further deteriorated with the increasing severity of HE and correlated cognitive dysfunction in cirrhotic patients.

## Introduction

Hepatic encephalopathy (HE) is a neuropsychiatric syndrome that develops in patients with severe liver diseases and/or portosystemic shunt surgery resulting from a serious complication of acute and chronic liver failure [1]. HE has been considered as a continuum of neurocognitive dysfunction: from minimal HE (MHE), the mildest manifestation of HE, to overt HE, and ultimately to coma and death [2]. MHE is defined as a subpopulation of cirrhotic patients without obvious clinical manifestation of HE but can be identified with neuropsychological tests, such as number connection test (NCT) and digit symbol test [DST] [1], [2]. In last decade, MHE has received broad attention because of its high prevalence, impaired driving skills [3]–[5], decreased quality of life [6], and increased falls [7]. The impaired abilities in MHE patients are attributed to cognitive dysfunctions affecting attention, memory, and fine motion.

Attention deficit is a fundamental aspect of HE [8]–[10]. Impairment of attention function is the earliest and most striking finding in cirrhotic patients which has been demonstrated by neuropsychological testing [8]–[10] and functional imaging [11]–[13]. In MHE patients, inability to sustain attention, decreased error detection, and psychomotor slowing have been widely reported in behavioral studies [8]–[10]. The attention deficits are considered to be due especially to dysfunction of anterior attention system, composed of the frontomedial and fronto-lateral cortex, and the anterior cingulate cortex (ACC), as proposed by Posner and Petersen [14]. The ACC is regarded as a core region involved in attention which severs both regular cognitive and emotional processing [15]. The ACC is also a core node of the default mode network [16]. Thus, the ACC is an important brain area that may be associated to the functional deficit of HE patients Many neuroimaging studies demonstrated the following: abnormal blood flow, neural activity, and metabolites of the ACC in cirrhotic patients with or without HE, using single photon emission computed tomography (SPECT) [17], positron emission tomography (PET) [18], magnetic resonance spectroscopy (MRS) [19], and functional MRI [11], [12], [20], [21]. Iwasa et al. [17] observed reduced blood flow in the ACC in most cirrhotic patients. They concluded that blood flow in the ACC as measured by SPECT may be an indicator of cerebral functional changes in patients with cirrhosis. In proton MRS investigation, lower ratios of choline (Cho)/Cr and myo-inositol (mIns)/Cr and a higher ratio of glutamine-glutamate (Glx)/Cr, were observed in the ACC in MHE patients [19]. Functional MRI studies reported by Zhang et al. [11] revealed that the activation in the ACC was decreased comparing to that of the control when patients with hepatic cirrhosis performed the incongruous color-naming task compared with controls. Furthermore, Hsu et al. [21] found that increased severity of HE was associated with significantly reduced node strength and integrated relative and absolute regional efficiency in the frontal and temporal cortices, including the ACC. Thus, the ACC seems to be an appropriate location for investigating functional connectivity changes in patients with cirrhosis. However, to our knowledge, the effect of cirrhosis on the functional connectivity involving ACC has not been investigated in patients. The purpose of this study was to evaluate the pattern of changes of functional connectivity in ACC of patients with cirrhosis and the correlation of functional connectivity changes with clinical markers, such as venous blood ammonia levels and neuropsychiatric tests, in order to investigate the role of changes in ACC functional connectivity in the development of HE.

## Materials and Methods

### Subjects

This retrospective study was approved by the Medical Research Ethics Committee of Jinling Hospital and Clinical School of Medical College at Nanjing University. The written informed consents were obtained from all subjects. Sixty seven cirrhotic patients (52 male, 15 female, mean age: 49.85±9.44 years) hospitalized at Jinling Hospital were included in this study. The inclusion criteria for recruitment of the patients were as follows: age of 18 years or older with clinically diagnosed cirrhosis, without clinical manifestation of overt HE, abnormal neuropsychological test scores, who could finish the MR exam without any MRI contraindication. Subjects were excluded from the study if they have any one of the following conditions: drug abuse history, brain lesions such as tumor, stroke assessed on basis of medical history and conventional MRI, or having head motion with translation more than 1.0 mm or rotation more than 1.0° during MR scanning.

Forty age-and gender-matched right-handed healthy controls from local community (30 male, 10 female, and mean age: 52.12±8.14 years) were recruited in this study. All healthy controls had no diseases of the liver or other systems, no abnormal findings in abdominal ultrasound scans and conventional brain MR imaging. All controls underwent neuropsychological tests before the MR scanning. No laboratory tests were performed; thus, these data were unavailable for them.

### Neuropsychological tests

The diagnosis of MHE was made according to the recommendation by the working party of 11^th^ World Congress of Gastroenterology in Vienna in 1998 [22]. The test battery includes number connecting test type-A (NCT-A) and digit symbol test (DST) which were recommended by the working party. NCT-A tests for psychomotor speed with the test score being the time the subject performs the test. The worse performance is indicated by a longer time for completion with the test. DST tests for psychomotor speed, attention, and visual memory, with the test score being the total number of correct sequential matching of symbols to numbers in a 90-second interval. When the scores of at least one test were beyond 2SD (standard deviation) of mean value of age-matched healthy controls, the cirrhotic patients were categorized as MHE.

### Laboratory Examinations

Laboratory tests including prothrombin time, protein metabolism tests, venous blood ammonia were performed on all patients to assess the severity of liver disease, within one week before MR scanning. The grade of hepatic function was determined according to the Child-Pugh score [23]. The score system consists of five variables, i.e., ascites, encephalopathy, prothrombin time, and serum levels of bilirubin and albumin. A assigned score ranges from 1 to 3 after counting for each variable.

### MRI data acquisition

MRI data were acquired on a 3 Tesla MR scanner (TIM Trio, Siemens Medical Solutions, Erlangen, Germany). All the patients and healthy controls were instructed to close their eyes but keep awake during the resting-state functional MR imaging examination. Foam pad was used to minimize the head motion of all subjects. High-resolution T1-weighted 3D anatomical images were first obtained in the sagittal orientation using a magnetization-prepared rapid gradient-echo sequence (TR/TE = 2300 ms/2.98 ms, flip angle = 9°, 191 slices, FOV = 256×256 mm^2^, acquisition matrix = 256×256, slice thickness = 1 mm). Functional images were then obtained with a single-shot, gradient-recalled echo planar imaging sequence (TR/TE = 2000 ms/30 ms, FOV = 240×240 mm^2^, flip angle = 90°, matrix = 64×64, voxel size = 3.75×3.75×4 mm^3^). A total of 250 brain volumes were collected, resulting in a total scan time of 500 s.

### Data preprocessing

Data were pre-processed using SPM8 software package (http://www.fil.ion.ucl.ac.uk/spm). The first 10 volumes were excluded for magnetization to reach equilibrium and the remaining 240 consecutive volumes were used for data analysis. Slice-timing adjustment and realignment for head-motion correction were performed. No translation or rotation parameters in any given data set exceeded 1.0 mm or 1.0°. We also evaluated the group differences in translation and rotation of head motion according to the following formula (1):
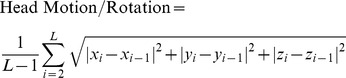
Where *L* is the length of the time series (*L* = 240 in this study), *xi*, *yi* and *zi* are translations/rotations at the *i*th time point in the *x*, *y* and *z* directions, respectively. The results showed that the patient and control groups had no significant differences in image quality (one-way analysis of variance [ANOVA]: *F* = 1.424, *P* = 0.245 for translational motion, and *F* = 0.477, *P* = 0.622 for rotational motion). The functional images were then spatially normalized to standard stereotaxic coordinates of the Montreal Neurological Institute (MNI) and resampled into voxel size of 3×3×3 mm^3^. They were then smoothed with an isotropic Gaussian kernel of 8 mm full width half maximum (FWHM) to decrease spatial noise. To further reduce the effects of confounding factors, which is unlikely involved in specific regional correlation, we also removed several sources of spurious variance by linear regression. These variances included six head motion parameters, average signals from cerebrospinal fluid, white matter, and whole brain. Then, the residual time series were band filtered (0.01–0.08 Hz) using the REST software (http://resting-fmri.sourceforge.net).

### Definition of seed regions

In individual rs-fMRI data analysis, we used the bilateral pregenual ACC as one single seed region because similar functional connectivity of the left and right pregenual ACC has been reported in a previous study [24]. WFU PickAtlas Tool Version 3.0 (http://fmri.wfubmc.edu/software/PickAtlas) [25], a toolbox which provided an atlas-based method of generating regions of interest (ROIs) was used to define the seed region of bilateral ACC. The ROI of bilateral ACC was defined using the automated anatomical labeling (AAL) atlas provided in this toolbox and was used to define the reference time series using the approach reported in previous rs-fMRI studies [26].

### Functional connectivity analysis

For each subject, a correlation map was produced by computing the cross-correlation coefficient (*R* score) between the reference time series of the seed region (bilateral ACC) and the time series of each voxel within the whole brain. Correlation coefficients were then converted to *Z* values using Fisher's *R*-to-*Z* transform to standardize the statistical analysis, since the correlation coefficient *R* is not normally distributed [27].

### Statistical analysis

#### Group analysis of the ACC functional connectivity

Within each group, a random effect one-sample *t* test was performed on an individual *Z* value map in a voxel-wise manner to determine brain regions showing significant functional connectivity to the seed region of the ACC. Significant thresholds were set at a corrected *P*<0.001 with multiple sample correction using false discovery rate (FDR) criterion [28] across the whole brain. An ANOVA test in a voxel-wise manner was then performed to determine differences of the ACC RSN between MHE, non-HE patients and healthy controls, with age and sex, and head motion as covariates. Statistical threshold was also set at *P*<0.05, FDR corrected.

### Pearson correlation analysis of the ACC functional connectivity

To investigate the association between the venous blood ammonia levels, neuropsychological performance and the ACC RSN in the MHE patients, a Pearson correlation analysis was performed between the *Z* value of the brain regions within the ACC RSN and the venous blood ammonia levels, neuropsychological test scores in cirrhotic patients in a voxel-wise way. Statistical threshold was set at *P*<0.05 (after FDR correction).

## Results

### Demographics and clinical data

Demographics and clinical data for all 107 participants were summarized in **Table 1**. There was no significant difference in gender or age between cirrhotic patients and healthy control groups (both *P*>0.05). However, cirrhotic patients demonstrated worse neuropsychological performance than healthy controls; they spent more time to complete the NCT-A (45.62±8.73 s) and had lower scores of DST (42.96±10.71 scores) (both *P*<0.05) compared with healthy controls (56.82±20.90 s for NCT-A and 34.41±12.11 scores for DST; **Table 1**).

**Table 1:** Demographics and clinical data of all cirrhotic patients and healthy controls.

Protocols	HC (n = 40)	NHE (n = 40)	MHE(n = 27)	*P* value
Sex (M/F)	30/10	32/8	20/7	0.82^a^
Age (±SD), y	52.12±8.14	46.35±9.66	55.04±6.28	0.001^b^
Venous blood ammonia (in umol/L)		47.44±32.72	62.26±28.60	0.119^c^
Child-Pugh scale				
A		28	12	
B		11	14	
C		1	1	
NCT-A	45.62±8.73	44.20±8.97	75.52±19.48	0.001^b^
DST	42.96±10.71	40.98±9.79	24.70±8.02	0.001^b^

Values are expressed as mean ± SD. HC = healthy control; NHE = non-hepatic encephalopathy; MHE = hepatic encephalopathy; NCT-A = number connecting-A; DST = digit symbol test.

a The *P* value for gender distribution in the three groups was obtained by chi-square test.

b The *P* value for age and neuropsychological tests difference among the three groups was obtained by one way ANOVA.

c The *P* value for blood ammonia in the two patient groups was obtained by two sample *t* test.

### Within-group Comparison of the ACC resting-state functional network

**Figure 1:**
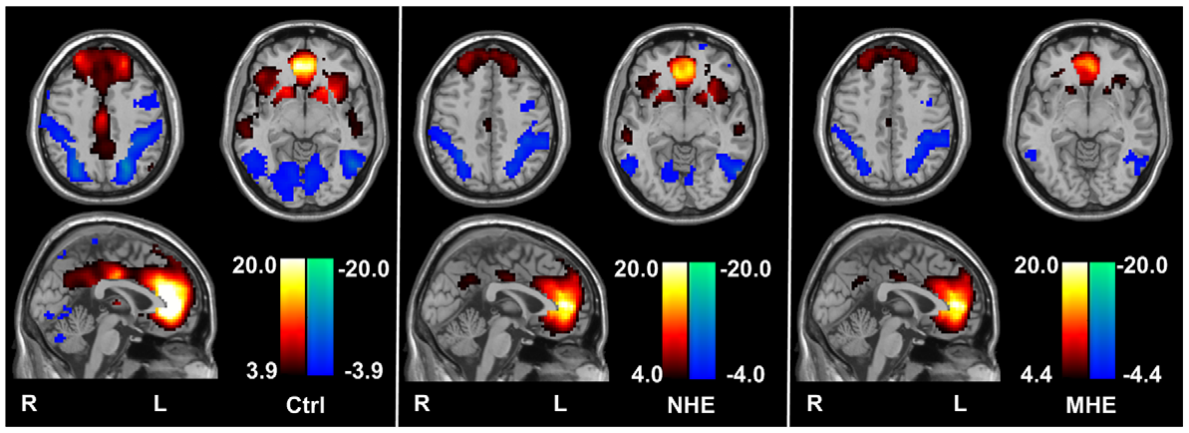
One sample t test result of the ACC functional connectivity for each subject group. In all subjects, the ACC positively correlates with the PCC/precuneus, lateral parietal cortex, medial prefrontal cortex, superior frontal gyrus, and the middle and inferior temporal gyri, the medial prefrontal cortex, orbitofrontal cortex, temporal lobe, insula, thalamus, caudate, putamen, and other cingulate subregions (*P*<0.05, FDR corrected). The ACC shows negative functional connectivity with the precentral and postcentral gyri, prefrontal cortex, parietal cortex, cuneus lobe, lingual and fusiform gyri (*P*<0.05, FDR corrected). ACC = anterior cingulate cortex; Contr = control; NHE = non-hepatic encephalopathy; MHE = minimal hepatic encephalopathy.

Similar functional connectivity patterns were observed in all subjects, although there is a decreased tendency of functional connectivity in the ACC of cirrhotic patients compared with healthy controls (*P*<0.05, FDR corrected) (**Fig. 1**). The functional connectivity patterns of ACC were very similar in the right and left hemispheres. In all subjects, functional connectivity of ACC positively correlated with the default mode network (DMN), including PCC/precuneus (Pcu), lateral parietal cortex, medial prefrontal cortex, superior frontal gyrus, and the middle and inferior temporal gyri (MTG/ITG). The affective network (AN) included the medial prefrontal cortex, orbitofrontal cortex, and temporal pole, the insula, thalamus, caudate and putamen. ACC was also positively correlated with all other cingulate subregions (*P*<0.05, FDR corrected) (**Fig. 1**). ACC of cirrhotic patients showed negative functional connectivity with the sensorimotor network (SMN), including the precentral and postcentral gyri; cognitive network (CN), including the dorsolateral prefrontal cortex (DLPFC), ventrolateral prefrontal cortex and dorsolateral parietal cortex; and visual network (VN), including the cuneus lobe, and lingual and fusiform gyri (*P*<0.05, FDR corrected) (**Fig. 1**).

### Inter-group comparisons of the ACC resting-state functional network

**Figure 2:**
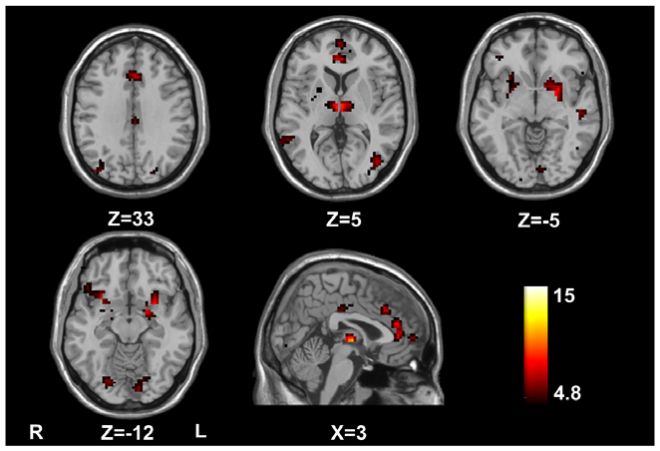
ANOVA test result of the ACC functional connectivity among three subject groups. ANOVA test result for the functional connectivity differences among healthy controls, non-HE patients, and MHE patients shows the significantly different functional connectivity in the following brain areas: bilateral ACC, middle frontal cortex, and middle cingulate cortex, bilateral superior temporal gyri/middle temporal gyri, thalami, putamen, insula, middle occipital gyrus, lingual gyrus, and left temporo-occipital cortex (*P*<0.05, FDR corrected). HE = hepatic encephalopathy; MHE = minimal hepatic encephalopathy; ACC = anterior cingulate cortex.

**Figure 3:**
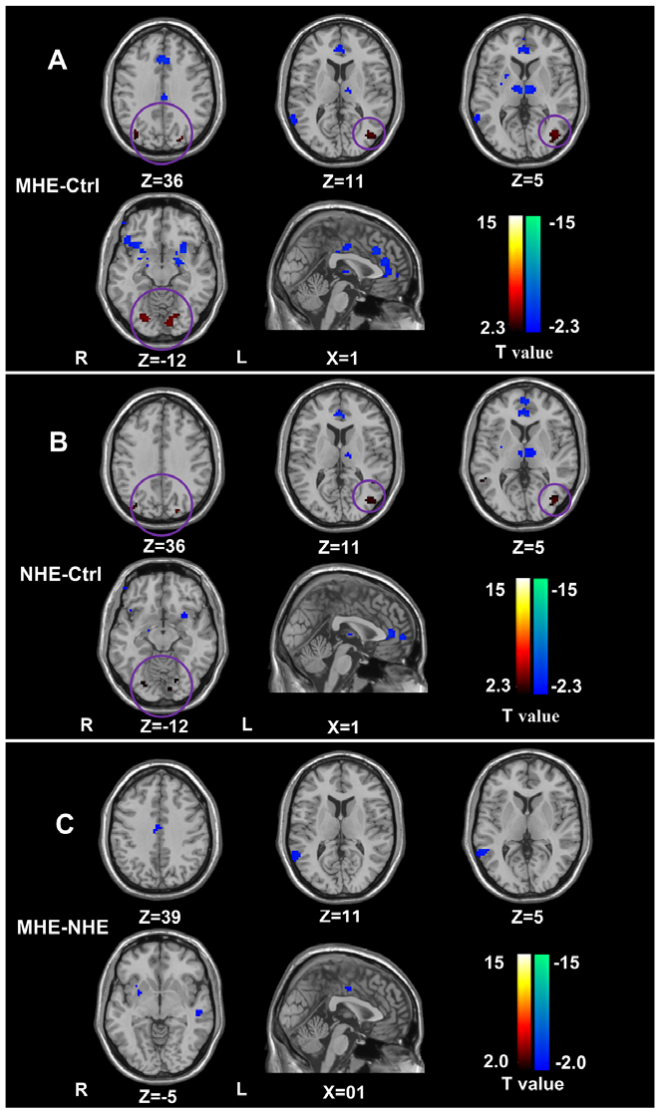
Inter-group analysis of the ACC functional connectivity among three subject groups. (A) MHE-control result. Compared with healthy controls, MHE patients show the decreased positive functional connectivity between the ACC seed and bilateral MFC/ACC, bilateral MCC, bilateral STG/MTG, bilateral thalami, bilateral putamen and bilateral insula; and decreased negative functional connectivity between the ACC seed and bilateral MOG and left temporo-occipital lobe and bilateral lingual gyri color coded dark red in circle (*P*<0.05, FDR corrected). (B) MHE-control result. Compared with healthy controls, non-HE patients show the decreased positive functional connectivity between the ACC seed and bilateral MFC/ACC, bilateral thalami, bilateral putamen and left insula; increased positive functional connectivity between the ACC seed and right MTG, and decreased negative functional connectivity between the ACC seed and right MOG, left temporo-occipital lobe color coded dark red in circle (*P*<0.05, FDR corrected). (C) MHE-non-HE result. Compared with non-HE patients, MHE patients show the decreased positive functional connectivity between the ACC seed and right MCC, bilateral STG/MTG and right putamen (*P*<0.05, FDR corrected). Contr = control; NHE = non-hepatic encephalopathy; MHE = minimal hepatic encephalopathy; ACC = anterior cingulate cortex; MFC = middle frontal cortex; MCC = middle cingulate cortex; STG = superior temporal gyrus; MTG = middle temporal gyrus; MOG = middle occipital gyrus.

The results from ANOVA revealed the differences in functional connectivity among healthy controls, non-HE patients, and MHE patients. **Figure 2** showed significantly different functional connectivity in the following brain areas: bilateral ACC, middle frontal cortex (MFC), middle cingulte cortex (MCC), bilateral superior temporal gyri/middle temporal gyri (STG/MTG), thalami, putamen, insula, middle occipital gyrus (MOG), lingual gyrus, and left temporo-occipital cortex (*P*<0.05, FDR corrected). To analyze the inter-group functional connectivity difference of the ACC, we also performed Post-hoc tests. Compared with healthy controls, MHE patients showed the decreased positive functional connectivity between the ACC seed and bilateral MFC/ACC, bilateral MCC, bilateral STG/MTG, bilateral thalami, bilateral putamen and bilateral insula. These patients also showed increased negative functional connectivity between the ACC seed and bilateral MOG and left temporo-occipital lobe and bilateral lingual gyri (*P*<0.05, FDR corrected) (**Table 2** **,** **Fig. 3A**). Compared with healthy controls, non-HE patients showed the decreased positive functional connectivity between the ACC seed and bilateral MFC/ACC, bilateral thalami, bilateral putamen and left insula. The non-HE patients also showed decreased negative functional connectivity between the ACC seed and right MOG, left temporo-occipital lobe, and increased positive functional connectivity between the ACC seed and right MTG (*P*<0.05, FDR corrected) (**Table 3** **,** **Fig. 3B**). No other group difference was found. Compared with non-HE patients, MHE patients only showed the decreased positive functional connectivity between the ACC seed and right MCC, bilateral STG/MTG and right putamen (*P*<0.05, FDR corrected) (**Table 4** **,** **Fig. 3C**). No difference of increased functional connectivity between patients and controls was noted. These findings indicated decreased ACC functional connectivity predominated with the increasing severity of HE.

**Table 2:** Regions showing abnormal functional connectivity with ACC between the MHE patients and healthy controls.

Regions	Hem	BA	MNI coordinates (mm)	ΔVol (mm^3^)	Maximal *t* value
			(x, y, z)		
**Decreased**					
ACC	L	32	−6,39,12	65	−2.52
ACC	R	32	3,42,12	24	−2.29
MFC	R/L	10	−3,51,0	10	−2.81
MCC	L	32/24	−9,30,30	45	−3.10
MCC	R	32/24	6,27,27	28	−3.14
STG/MTG	L	22/21	−48,−21,−6	29	−3.25
STG/MTG	R	22/21	61,−54,12	45	−1.96
Thalamus	L	…	−18,−18,6	59	−2.41
Thalamus	R	…	15,−12,6	30	−2.86
Putamen	L	…	−18,15,−6	60	−2.61
Putamen	R	…	30,−6,0	54	−2.57
Insula	L	13	−30,12,−15	30	−2.60
Insula	R	13/47	39,18,−12	35	−2.75
MOG#	R	19	15,−78,51	28	−2.99
temporo-occipital#	L	9/6	−42,−72,6	79	−3.93
Lingual gyrus#	L	18	−12,−87,−12	54	−4.22
Lingual gyrus#	R	18	21,−78,−15	35	−4.38

Positive sign represents increased functional connectivity, and negative sign represents decreased functional connectivity. MHE = hepatic encephalopathy; FDR = false discovery rate; Hem = hemisphere; BA = Brodmann's area; MNI = Montreal Neurological Institute; ΔVol: volume difference; ACC = anterior cingulate cortex; MCC = middle cingulate cortex; STG = superior temporal gyrus; MTG = middle temporal gyrus; MOG = middle occipital gyrus.

# Indicates reduced negative functional connectivity in the patients.

**Table 3:** Regions showing different functional connectivity with ACC between the NHE patients and healthy controls.

Regions	Hem	BA	MNI coordinates (mm)	ΔVol (mm^3^)	Maximal *t* value
			(x, y, z)		
**Decreased**					
ACC	L	32	−6,42,3	37	−2.46
ACC	R	32	3,45,6	14	−2.53
MFC	R/L	10	−3,54,6	15	−2.86
Thalamus	L	…	−6,−18,6	54	−2.41
Thalamus	R	…	3,−15,6	11	−2.37
Putamen	L	…	−18,6,−6	10	−2.60
Putamen	R	…	30,−3,−3	10	−2.43
Insula	L	13	−21,9,−15	11	−2.54
MOG#	L	19	−6,−81,84	19	−2.54
MOG#	R	19	15,−81,48	35	−4.45
temporo-occipital#	L	9/6	−42,−75,6	56	−3.37
**Increased**					
MTG	R	21	54,−51,6	10	+3.18

Positive sign represents increased functional connectivity, and negative sign represents decreased functional connectivity. NHE = non-hepatic encephalopathy; FDR = false discovery rate; Hem = hemisphere; BA = Brodmann's area; MNI = Montreal Neurological Institute; ΔVol: volume difference; ACC = anterior cingulate cortex; MFC = medial frontal cortex; MOG = middle occipital gyrus; MTG = middle temporal gyrus;

# Indicates reduced negative functional connectivity in the patients.

**Table 4:** Regions showing different functional connectivity with ACC between the MHE and NHE patients.

Regions	Hem	BA	MNI coordinates (mm)	ΔVol (mm^3^)	Maximal *t* value
			(x, y, z)		
**Decreased**					
MCC	R	32/24	0,9,42	12	−3.11
STG/MTG	L	22/21	−54,−24,3	23	−3.30
STG/MTG	R	22/21	57,−51,6	60	−4.22
Putamen	R	…	27,12,−9	10	−3.01

Negative sign represents decreased functional connectivity. MHE = hepatic encephalopathy; NHE = non-hepatic encephalopathy; FDR = false discovery rate; Hem = hemisphere; BA = Brodmann's area; MNI = Montreal Neurological Institute; ΔVol: volume difference; ACC = anterior cingulate cortex; MCC = middle cingulate cortex; STG = superior temporal gyrus; MTG = middle temporal gyrus.

### Correlations of ACC functional connectivity with digital symbol test and number connection test

**Figure 4:**
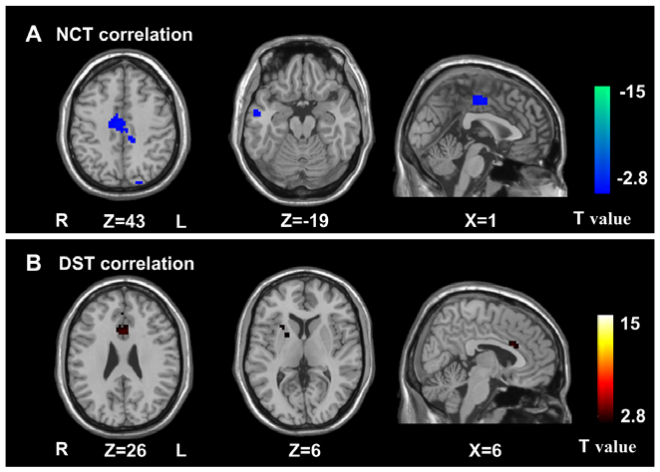
Pearson correlation analysis of the ACC functional connectivity. Pearson correlation analyses reveals that NCT-A scores negatively correlate with ACC functional connectivity in the bilateral MCC, right MTG (*P*<0.05, FDR corrected) (**upper panel**); that DST scores positively correlate with bilateral ACC and the right putamen (*P*<0.05, FDR corrected) (**low panel**). NCT-A = number connection test type A; ACC = anterior cingulate cortex; MCC = middle cingulate cortex; MTG = middle temporal gyrus; DST = digit symbol test.

Pearson correlation analyses revealed that NCT-A scores negatively correlated with ACC functional connectivity in the bilateral MCC, right MTG (*P*<0.05, FDR corrected) (**Fig. 4A**); DST scores positively correlated with bilateral ACC and right putamen (*P*<0.05, FDR corrected) (**Fig. 4B**) in cirrhotic patient group. No correlation was found between venous blood ammonia levels and functional connectivity of the ACC in cirrhotic patients.

## Discussion

This study found disrupted ACC functional connectivity in cirrhotic patients without overt HE which further deteriorated with the increasing severity of HE. Disrupted functional connectivity in the ACC in cirrhotic patients correlated with cognitive dysfunctions measured by NCT-A and DST. To the best of our knowledge, this is the first report on disrupted ACC functional connectivity in cirrhotic patients without overt HE mapped by resting state fMRI.

In this study, the ACC functional connectivity patterns mapped in healthy controls, non-HE patients, and MHE patients were in line with those reported in the previous study [24]. The ACC functional connectivity positively correlated with the DMN, AN, and brain regions that process different aspects of emotionally salient stimuli, the thalamus, caudate, putamen and all other cingulate subregions. The ACC functional connectivity negatively correlated with the SMN, CN, VN. All subjects had the similar functional connectivity patterns of the ACC, however, cirrhotic patients had decreased tendency of functional connectivity of the ACC. This suggests that our findings on the ACC functional connectivity are reliable.

In this study, we found the ACC functional connectivity was predominantly decreased from non-HE to MHE, especially in the right MCC, bilateral STG/MTG, and right putamen. The MCC is activated during a variety of cognitive tasks including conflict monitoring, error detection, response selection, and attention control [29]–[32]. FMRI studies showed the MCC positively connected with the DLPFC which showed decreased functional connectivity in this study and our previous DMN study [20]. The anatomic studies demonstrated the reciprocal connections between these two brain regions [33]. The MCC also negatively correlated with NCT-A scores. NCT-A test for psychomotor speed [2] has been considered as the most sensitive diagnostic test for the detection of MHE. This indicated that MCC functional connectivity abnormality could reflect neurocognitive deficits in MHE patients. We found that bilateral STG/MTG functional connectivities were significantly decreased in MHE patients compared with non-HE patients. Recently, Montoliu et al. [34] found a focal thinning of STG in MHE patients compared with non-HE patients and controls using a cortical-based analysis technique. STG contains the primary auditory cortex, which is involved in auditory processing, including language, but also has been implicated as a critical structure in social cognition [35], [36]. Previous studies showed that cognitive auditory components with neural sources out of the primary auditory cortex were helpful for diagnosis of MHE [35]. Interestingly, the STG functional connectivity was increased in non-HE patients compared with controls, while it was decreased in MHE patients compared with non-HE patients and controls, indicating STG can be a specific brain region in the development of MHE. The non-HE patients may require more functional connectivity of STG to maintain cognitive balance, but it was further decreased with the increasing severity of HE. The negative correlation of STG functional connectivity with NCT-A scores also supported the role of STG in cognitive dysfunction in cirrhotic patients. However, the precise role of STG in the diagnosis of MHE remains unclear, and further investigation is needed in the future. We observed that DST scores positively correlated with bilateral ACC and right putamen. The putamen is one of the structures that comprise the basal ganglia, which is connected to the substantia nigra and globus pallidus through various pathways. The main function of the putamen is to regulate movements and influence various types of learning [37], [38]. In addition, the thalamus and insular functional connectivity were decreased in non-HE and MHE patients compared with controls. The disrupted functional connectivity of these cortical and subcortical networks, including the insula, thalamus and putamen may have an adverse effect on cognitive function in MHE patients. The above-mentioned brain areas are composed of anterior attention system proposed by Posner and Peterson [14]; these decreased functional connectivities of the above-mentioned brain areas can better interpret neurocognitive abnormality in MHE patients.

Although no difference was found between MHE and non-HE patients in cirrhotic patients compared with healthy controls, we also observed increased negative functional connectivity between the ACC seed and MOG, lingual gyrus and temporo-occipital junction, and the components of posterior attention system. This latter observation indicates that the increased negative functional connectivity between the ACC seed and these brain areas is not specific for the development of MHE. Our findings were supported by one previous behavioral study which concluded that the anterior attention system is more sensitive than the posterior attention system to the early stages of hepatic encephalopathy [10]. The above-mentioned brain areas composing of visual spatial information processing network are involved in attention information adjustment and orienting [39], [40]. It can also be interpreted as a compensatory mechanism for abnormal anterior attention system in MHE patients because the posterior attention system is thought to operate upon the ventral pathway during tasks requiring detailed processing of objects [14]. However, further investigations are needed for the role of increased negative functional connectivity between the ACC seed and MOG, lingual gyrus, and temporo-occipital cortex in cirrhotic patients in the development of OHE.

Our study has several limitations. First, our study is confined to analyze the pregenual ACC rather than all subregions of the ACC or whole brain functional network in cirrhotic patients without overt HE. The effects of HE may be more global. Other additional networks or whole brain network should be performed in future. For example, we observed functional connectivity of DMN was impaired in cirrhotic patients [20], [41]. Second, although we did observe the functional connectivity changes of the ACC during the development of MHE, the functional connectivity changes of the ACC in overt HE was not further investigated because of severity of overt HE. Third, we choose bilateral pregenual ACC as a seed region rather than left or right subregions of ACC in this study because one previous study demonstrated that the subregions of the ACC had similar functional connectivity. Additionally, side difference of the ACC in cirrhotic patients did not perform although one previous study demonstrated no asymmetry was observed in healthy controls [24]. Fourth, our study results could not exclude the interference of structural changes in the ACC. Future study linking structural and functional networks of the ACC is warranted to confirm the finding of this study.

Notwithstanding these limitations, our preliminary experimental study demonstrates that disrupted ACC functional connectivity existed in cirrhotic patients without overt HE. This further deteriorated with the increasing severity of HE and correlated cognitive dysfunction in cirrhotic patients. The disrupted ACC functional connectivity in cirrhotic patients appears imbalance of predominately decreased functional connectivity of anterior attention system and posterior attention system. This study shed on light for understanding neurocognitive basis of attention deficits in cirrhotic patients.
